# A silent myocardial infarction in the diabetes outpatient clinic: case report and review of the literature

**DOI:** 10.1530/EDM-13-0058

**Published:** 2013-10-28

**Authors:** M S Draman, H Thabit, T J Kiernan, J O'Neill, S Sreenan, J H McDermott

**Affiliations:** Department of EndocrinologyRoyal College of Surgeons in Ireland, Connolly Hospital, BlanchardstownDublinIreland; 1 Department of CardiologyUniversity of LimerickLimerickIreland

## Abstract

Silent myocardial ischaemia (SMI), defined as objective evidence of myocardial ischaemia in the absence of symptoms, has important clinical implications for the patient with coronary artery disease. We present a dramatic case of SMI in a diabetes patient who attended annual review clinic with ST elevation myocardial infarction. His troponin was normal on admission but raised to 10.7 ng/ml (normal <0.5) when repeated the next day. His angiogram showed diffused coronary artery disease. We here discuss the implications of silent ischaemia for the patient and for the physician caring for patients with diabetes.

**Learning points:**

- Silent myocardial ischaemia (SMI) is an important clinical entity.
- SMI is common and occurs with increased frequency in patients with diabetes.
- SMI is an independent predictor of mortality.
- Recognition may lead to early intervention.

## Background

Chest pain is a frightening symptom that typically alerts both the patient and the physician to the likelihood of underlying coronary artery disease. Absence of chest pain despite significant myocardial ischaemia is an important clinical entity. Such silent myocardial ischaemia (SMI), defined as objective evidence of myocardial ischaemia in the absence of symptoms, has important clinical implications for the patient with coronary artery disease. We present a dramatic case of SMI in a patient with diabetes, describe the aetiology of the condition and discuss the implications of silent ischaemia for the patient and for the physician caring for patients with diabetes.

## Case presentation

A 62-year-old man attended the diabetes outpatient clinic for his annual review visit. He had been diagnosed with type 2 diabetes mellitus 6 years earlier. His diabetes was complicated by diabetic nephropathy, background diabetic retinopathy and peripheral neuropathy manifested by reduced vibration sense in both feet. He also suffered from hypertension and dyslipidaemia. His medications at the time of review included an angiotensin converting enzyme inhibitor, an α-blocker, a statin, metformin and aspirin.

He was symptomatically well. Physical examination revealed a blood pressure of 155/83 mmHg, reduced vibration sense in his feet and palpable peripheral pulses. Laboratory tests revealed a HbA1c of 6.1%, lipid profile within goal range with total cholesterol 3.4 mmol/l, triglyceride 1.10 mmol/l, HDL 1.64 mmol/l and LDL 1.29 mmol/l. Serum creatinine was elevated at 120 mmol/l (58–110) and 24-h urinary protein 1.13 g.

**Figure 1:**
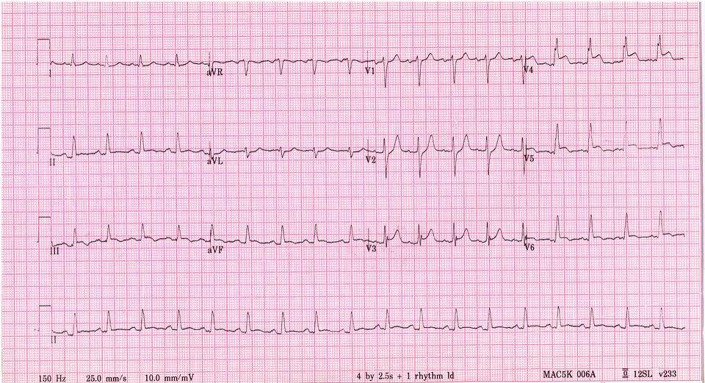
ECG showing anterolateral ST elevation.

After his physician review was complete, an electrocardiogram (ECG) was performed as part of his routine annual assessment (Fig. 1). The patient was recalled to the clinic room for further review as a result of the ECG findings. On questioning, he denied chest, arm or jaw pain or discomfort; shortness of breath; palpitations; nausea; indigestion or sweating. In view of the ECG changes, he was admitted for investigation and treatment of a suspected myocardial infarction.

## Investigation

The patient remained symptomatically well throughout his hospital stay. Further investigations revealed a normal troponin I of 0.3 ng/ml on the day of admission, rising to 10.7 ng/ml (normal <0.5) when repeated the next day. Creatine phosphokinase was normal at 100 U/l on admission, rising to 273 U/l (55–270) the next day, with an MB fraction of 17% (0–4). Echocardiogram demonstrated a regional wall motion abnormality, with apical hypokinesis. A coronary angiogram was performed and revealed diffuse coronary artery disease with a small non-dominant right coronary artery, a distal 100% stenosis of the left anterior descending artery and a 100% obtuse marginal stenosis. The operator was not able to cross the occlusion and decision was made to treat him medically.

**Figure 2:**
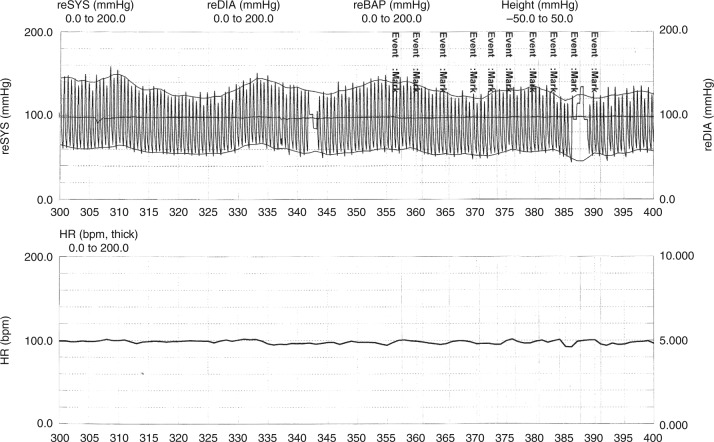
Absent heart rate response to controlled deep breathing (six breaths per minute). Event mark: recording during controlled deep breathing.

**Figure 3:**
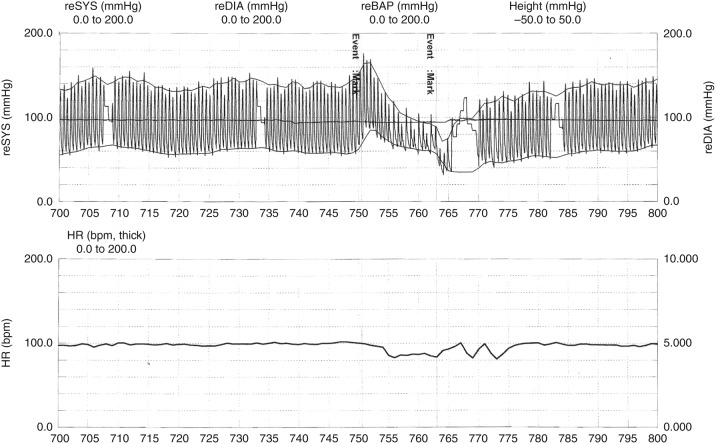
Diminished heart rate response to phases II and IV of Valsalva manoeuvre. Event mark: the beginning of the test.

**Figure 4:**
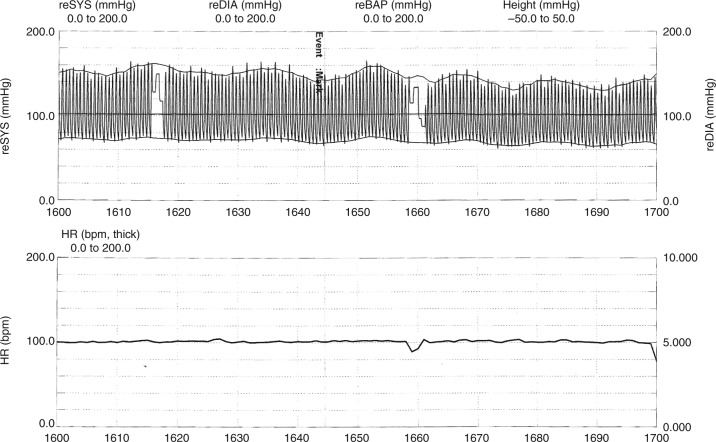
Absent heart rate response to tilting up on tilt table test at 1 min. Note absent postural drop with absent reflex tachycardia. Event mark: the beginning of tilting up.

In view of the silent nature of the patient's myocardial infarction, and the finding of peripheral neuropathy on examination, autonomic function testing was performed. This shows absence of heart rate variability in deep breathing, phase II and IV Valsalva manoeuvre response and poor heart rate response on tilt up test. This is consistent with a diagnosis of autonomic neuropathy (Figs 2, 3 and 4).

## Treatment

Patient was treated medically with aspirin 75 mg, clopidogrel 75 mg and bisoprolol 5 mg once daily in addition to his current medications and arranged to undergo cardiac rehabilitation programme.

## Outcome and follow-up

Patient was doing well following discharge and completed cardiac rehabilitation programme successfully. Unfortunately, the patient subsequently passed away ∼1 year following the event.

## Discussion

### Prevalence of SMI

Estimates of the prevalence of SMI vary widely between reports to a large degree depending on the definition of silent ischaemia used, the mode of ischaemia detection utilized and the population under study. In a cohort of the Framingham study (1), patients with a normal ECG and with no evidence of coronary artery disease at baseline were followed for 30 years, with biennial ECG and clinical review. More than one in four of all myocardial infarctions discovered had been previously undetected, and almost half of these infarctions were silent. The discovery of previously unrecognized ischaemia does not necessarily imply that the ischaemia was silent – perhaps the ischaemia was unrecognized as patients had not exercised sufficiently in their daily lives to elicit symptoms of ischaemia or had misinterpreted atypical angina.

The concept that patients with diabetes have a higher prevalence of SMI than the general population was suggested by early clinical and autopsy studies and has since become accepted teaching. Many more recent large studies have appeared to confirm the high prevalence of silent ischaemia in diabetes but have been somewhat limited by the varying definitions of SMI between studies. The Milan Study on Atherosclerosis and Diabetes (2) attempted to determine the prevalence of unrecognized SMI in patients with diabetes but without known coronary artery disease. Nine hundred and twenty-five patients with type 2 diabetes underwent exercise stress test (EST) followed by exercise thallium scintigraphy if the EST was abnormal. Of these, 12.1% had abnormal stress test alone and 6.4% of patients had an abnormal response to both tests, indicating a high likelihood of previous undetected myocardial ischaemia, a figure roughly three times that found in the general population. A higher figure was quoted in Detection of SMI in Asymptomatic Diabetic Subjects (DIAD) study in 2004 (3), which found a prevalence of silent ischaemia of 22% in patients with type 2 diabetes and no known coronary artery disease using EST and adenosine technetium-99m sestamibi single-photon emission-computed tomography myocardial perfusion imaging (MPI).

### Mechanism

Why might patients with diabetes have a higher prevalence of SMI than the general population? Several explanations are possible (including a different threshold of pain sensitivity or psychological denial) but cardiac autonomic neuropathy almost certainly plays an important role, potentially involving dysfunction at varying levels – from the pain receptors, afferent neurons or gating mechanisms to the supratentorial translation of ischaemia into pain. Many years ago, an autopsy study in a diabetic patient who had silent infarction found pathologic changes in cardiac afferent neurons consistent with a neuropathy (4), and the prolonged anginal threshold reported in patients with DM has been found to occur in association with reduced heart rate variability (5), an early sign of cardiac autonomic nerve dysfunction (CAN). The failure to develop angina at the onset of ischaemia will have a permissive effect on the diabetic subjects' exercise tolerance: they are able to exercise longer in the absence of chest pain and at risk of developing more severe ischaemia as a result.

These and other studies certainly suggest an association between CAN and silent ischaemia in patients with diabetes. A study by Langer *et al*. (6) attempted to demonstrate direct causation by examining cardiac innervation using metaiodobenzylguanidine imaging in patients with diabetes and no history of cardiovascular disease. Patients with DM had significant reductions in MIBG uptake compared with healthy controls and those patients with diabetes and SMI had greater abnormalities, further supporting the suggestion that abnormalities in myocardial pain perception in patients with diabetes are directly linked to sympathetic denervation.

### Prognosis

Direct evidence of the important prognostic implications of SMI does exist. In a study by Gottlieb *et al*. (7), the presence of episodes of myocardial ischaemia on ECG monitoring, the majority of which were silent, remained an independent predictor of mortality after adjustment for other known prognostic indicators. The possible consequences of SMI for the patient are manifold and are starkly illustrated by our case. If our patient had not been at our clinic and had not had a routine ECG performed on the day in question, his acute myocardial infarction would have gone unrecognized. This would have delayed the institution of medications proven to reduce mortality post-infarction, such as β-blockers and heparin. He would also have spent the immediate post-infarct period at his normal activities (and at risk of an out-of-hospital post-infarct arrhythmia) rather than in a monitored coronary care bed. It is well recognized that patients with diabetes have more advanced coronary artery disease at the time of initial presentation than their non-diabetic counterparts and are more likely to have multi-vessel disease. It is tempting to speculate that SMI, by causing a delay in presentation, may play a role in this.

### Screening

The high prevalence of cardiovascular disease combined with the apparently higher prevalence of SMI in patients with diabetes, and the possible consequences of undetected myocardial ischaemia as outlined above, would seem to strongly favour a screening programme for detection of asymptomatic coronary artery disease in the diabetic population. Screening remains a contentious issue, however. First, no screening test demonstrates sufficient specificity to avoid the need for invasive tests if the initial screening test is positive. Secondly, the benefit of detecting underlying asymptomatic coronary artery disease in patients who have not had an infarct remains unclear. The American Diabetes Association (ADA) recommends that diabetes should be considered as a coronary artery disease equivalent and the risk factors for coronary artery disease should be treated aggressively in a patient with diabetes whether or not he or she is known to have coronary artery disease. Therefore, while detection of SMI in a patient with diabetes may lead to further measures such as attempted revascularization or institution of β-blockers, direct evidence that these measures improve prognosis in patients with diabetes who have not had a myocardial infarction and who are asymptomatic is lacking.

In 2009, the DIAD group (8) published the first large-scale (1123 patients) prospective randomized controlled study of screening for SMI (with MPI) vs no screening. Patients were followed for 5 years, and screening had no effect on the likelihood of developing a cardiovascular event at follow-up. The Bypass Angioplasty Revascularization Investigation (BARI) 2 Diabetes Trial did not show any difference in cardiovascular outcome in patients treated with coronary revascularization compared with intensive medical therapy alone. This suggests that the hypothetical benefit of screening patients needing revascularization as opposed to optimum medical therapy may not be real. Among a subgroup of patients in the BARI 2 trial for whom coronary artery bypass grafting was deemed to be appropriate, however, the revascularization strategy did reduce the rate of cardiovascular events (9). At present, the ADA (10) recommends that screening for occult myocardial ischaemia should only be performed in patients with typical or atypical cardiac symptoms and an abnormal resting ECG. This represents a change in policy from the previous ADA recommendation to screen all patients with diabetes who have two or more additional risk factors for cardiovascular disease.

In our outpatient department, all patients with diabetes have a screening ECG performed at their annual review visit and this practice appears sensible. An ECG is an inexpensive test, and although not sensitive for lesser degrees of ischaemia, an abnormal ECG has a high specificity for detection of prior myocardial infarction. Unlike the case with lesser degrees of silent ischaemia, revascularization and administration of β-blockers have been proven to be of benefit in patients with a prior myocardial infarction; therefore, detection of a new ECG abnormality will almost certainly result in interventions that can improve prognosis.

### Conclusion

SMI is a common occurrence and appears to occur with increased frequency in patients with diabetes, likely as a result of cardiac autonomic dysfunction. The presence of SMI has important implications for the patient, as dramatically illustrated by the case we describe. Physicians caring for patients with diabetes should be alert to the possibility of silent ischaemia.

## Patient consent

Unfortunately, since the writing of this article, the patient has passed away.

## Author contribution statement

M S Draman, H Thabit and J H McDermott were involved in drafting of the manuscript; T J Kiernan, J O'Neill, S Sreenan and J H McDermott were involved in critical revision of the manuscript; and M S Draman, H Thabit, T J Kiernan, J O'Neill, S Sreenan and J H McDermott were the patient's physicians.
